# Age-Dependent Risk Factors in Pediatric Sleep-Disordered Breathing: A Large-Scale Cross-Sectional Study

**DOI:** 10.3390/medicina62040707

**Published:** 2026-04-07

**Authors:** Gintare Oboleviciene, Viktorija Mejeryte, Norvile Jotautaite, Egle Rinkeviciene, Inga Katiniene, Vaidotas Gurskis, Rimantas Kevalas, Valdone Miseviciene

**Affiliations:** Pediatric Department, Medical Academy, Lithuanian University of Health Sciences, Eiveniu Str. 2, LT-50103 Kaunas, Lithuania; viktorija.mejeryte@lsmu.lt (V.M.); vaidotas.gurskis@lsmu.lt (V.G.); rimantas.kevalas@lsmu.lt (R.K.); valdone.miseviciene@lsmu.lt (V.M.)

**Keywords:** sleep-related breathing disorders, pediatric sleep apnea, risk factors in children, prevalence of SRBDs, Pediatric Sleep Questionnaire

## Abstract

*Background and Objectives*: Sleep-related breathing disorders (SRBDs) are common but often underdiagnosed in children. Early identification is essential, as untreated pediatric SRBDs can lead to cognitive, metabolic, and cardiovascular complications. This study aimed to estimate the prevalence of children at risk for SRBDs, defined as those screening positive based on Pediatric Sleep Questionnaire (PSQ) scores, and to analyze the association with potential risk factors in the general pediatric population of Lithuania. *Materials and Methods*: This cross-sectional study included 1929 children aged 2–17 years. Parents completed a questionnaire covering demographics, health status, and the PSQ. A validated Lithuanian version of the PSQ was used, with ≥8 (40%) positive responses indicating suspected SRBDs. *Results*: Overall, 14.9% of children were suspected of having SRBDs, with the highest prevalence among those aged 7–11 years (17.5%), followed by 2–6 years (14.9%) and ≥12 years (12.6%) (*p* = 0.032). In preschoolers (2–6 years), the strongest predictors were attention deficit hyperactivity disorder (ADHD; *p* < 0.001, OR 4.456, 95% CI 1.992–9.968) and allergic rhinitis (*p* < 0.001, OR 2.268, 95% CI 1.433–3.591). In children aged 7–11 years, endocrine diseases showed the strongest association (*p* < 0.001, OR 13.366, 95% CI 2.914–61.313), followed by ADHD (*p* = 0.001, OR 5.693, 95% CI 1.981–16.363) and adenotonsillar hypertrophy (*p* < 0.001, OR 3.079, 95% CI 1.839–5.156). In adolescents (≥12 years), SRBDs were primarily associated with ADHD (*p* < 0.001, OR 17.513, 95% CI 9.597–31.961) and endocrine diseases (*p* < 0.001, OR 6.214, 95% CI 2.965–13.020), while obesity remained significant (*p* < 0.001, OR 3.400, 95% CI 2.106–5.489). *Conclusions*: Approximately 15% of Lithuanian children were at risk for SRBDs. Risk factors differed by age: adenotonsillar hypertrophy in school-aged children, allergic rhinitis in preschoolers, and obesity in adolescents, whereas ADHD was associated across age groups.

## 1. Introduction

Sleep-related breathing disorders (SRBDs) are characterized by abnormal respiration during sleep and include snoring, obstructive sleep apnea (OSA), central sleep apnea, and sleep-related hypoventilation [1,2,3]. OSA is a prevalent disorder in the pediatric population and the second most common chronic health condition after asthma [4]. However, it remains an underdiagnosed condition in children, as diagnostic approaches are often based on criteria designed for adults [5]. Pediatric OSA differs from adult OSA in its etiology, clinical presentation, and consequences [5,6]. Untreated pediatric OSA is associated with behavioral disorders, lower learning abilities, growth disturbances, obesity, and cardiovascular morbidities, which can result in long-term health complications, including an elevated risk of chronic cardiovascular diseases, metabolic disorders, and cognitive impairments in adulthood [6,7]. Consequently, early recognition and treatment of pediatric OSA are important for ensuring a healthier future population [6].

In-laboratory polysomnography (PSG) is the gold standard for diagnosing SRBDs [2,8,9]. However, sleep specialists face many challenges in performing the test due to children’s limited ability to cooperate with the setup [10]. Additionally, PSG remains a high-cost test with limited accessibility in many regions [9]. Therefore, few questionnaires have been developed to identify children with possible SRBDs [1,7]. Among these, the Pediatric Sleep Questionnaire (PSQ) created by Chervin et al. has shown the highest sensitivity (85%) and specificity (87%) [1,7,11]. The PSQ is a valid and reliable tool for children aged 2–18 years and includes 22 questions regarding snoring, breathing problems, sleepiness, behavioral problems, and other symptoms of OSA [11]. More than eight (33%) positive responses suggest an SRBD diagnosis [11]. However, the PSQ is a screening tool rather than a definitive diagnostic method for SRBDs in children [1,7].

The prevalence of OSA among children ranges from 0.7–13% in the general pediatric population [1,7,9]. The heterogeneity of prevalence in different regions is attributed to differences in methodologies and diagnostic criteria of OSA in children [7,9]. Additionally, racial, ethnic, and socioeconomic disparities in pediatric SRBDs should also be considered as factors affecting the prevalence rate [4]. Although PSG was developed almost 50 years ago [12], the prevalence of SRBDs in various regions, especially in the pediatric population, remains unknown. This study aimed to estimate the prevalence of children at risk for SRBDs, defined as those screening positive based on PSQ scores, and analyze the association with potential risk factors in the general pediatric population of Lithuania.

To our knowledge, this is the first study to examine the occurrence and clinical aspects of SRBDs in a pediatric population within the Baltic States.

## 2. Materials and Methods

### 2.1. Study Setting

This prospective cross-sectional study, involving children aged 2–17 years, was conducted between November 2021 and February 2023. This age range was selected as the PSQ is designed for use in this age group. We collected data through a survey administered to parents of children attending educational institutions in Lithuania. In total, 286 educational institutions across all Lithuanian regions participated in the study, including 96 kindergartens and 170 schools.

This study was reviewed by the Kaunas Regional Bioethics Committee and was deemed not to require ethical approval under the applicable regulations and guidelines. Participants (parents or legal guardians) received detailed information about the objectives and procedures of the study and were clearly informed that their participation would be anonymous. Only anonymous responses were collected from those who voluntarily agreed to participate. No personally identifiable information was obtained. Informed consent was provided by each participant through voluntary completion of the survey. The study adhered to ethical research principles, including voluntary participation, informed consent, and full anonymity of participants.

### 2.2. The Survey

The survey consisted of three parts. The first part of the survey included open-ended questions about the children’s age, height, weight, and frequency of infectious respiratory tract diseases during the year. We calculated body mass index (BMI) based on parent-reported height and weight. Overweight and obesity were defined as BMI-for-age growth chart values ≥85th and ≥95th percentiles, respectively. The second part included closed questions about the children’s health, including SRBDs symptoms; diagnosed tonsillar or adenoidal hypertrophy; past surgeries; allergic diseases; mental illness (ADHD); endocrine, neuromuscular, and cardiovascular diseases; and sleep hygiene. Parents provided subjective reports of excessive daytime sleepiness (EDS) and other symptoms. The third part of the survey included the PSQ by Chervin. The validated Lithuanian version of the PSQ, developed using a forward–backward translation process and demonstrating good psychometric properties, was used in this study [13]. The entire survey is provided in the Supplementary Material. Based on the Lithuanian validation study, a cutoff score of ≥8 positive responses (≥40%) was applied, differing from the original PSQ threshold of ≥33%. Accordingly, participants were divided into two groups according to their PSQ results: children with ≥8 positive answers were assigned to the suspected SRBD group, whereas those with <8 positive answers were assigned to the control group. For subgroup analysis, participants were additionally categorized into three age groups (2–6, 7–11, and ≥12 years), corresponding to preschool, primary, and secondary school developmental stages.

### 2.3. Statistical Analyses

Statistical analyses were conducted using SPSS software (version 29.0). Study groups were compared using chi-square analysis and the Kruskal–Wallis test as appropriate. Fisher’s exact test was applied to evaluate associations between categorical variables when the expected cell counts were less than 5. Descriptive statistics were used to calculate the means, medians, and confidence intervals (CIs) of continuous variables. Multivariable binary logistic regression analysis was performed to identify factors independently associated with suspected SRBDs. The backward stepwise likelihood ratio method was applied for variable selection in the logistic regression analysis, with initial variables selected based on clinical relevance and prior literature. To address the imbalance in the dataset, inverse probability weighting was applied based on the prevalence of the outcome. Odds ratios (ORs) and 95% CIs were also determined. Model discrimination was assessed by receiver operating characteristic (ROC) curve analysis, with calculation of the area under the curve (AUC) and corresponding 95% CI. Statistical significance was set at *p* < 0.05.

## 3. Results

Out of the 1983 collected responses, 1929 were included in the study. A total of 54 responses were excluded because the children did not meet the age criteria or the survey was filled out insufficiently or incorrectly. The study collected responses from 945 females and 979 males; five parents did not assign a specific gender to their children. The study comprised 544 children aged 2–6 years (28.2%), 676 aged 7–11 years (35.0%), and 709 aged ≥12 years (36.8%). No significant differences were observed between the age groups and sexes of the study population (*p* = 0.347).

Of all the respondents, 14.9% (*n* = 244) were suspected of having an SRBD. No significant differences in age (*p* = 0.142), sex (*p* = 0.485), or BMI (*p* = 0.072) were observed between the suspected SRBD and control groups. In the age group analysis, SRBDs were more commonly suspected in children aged 7–11 years compared with those aged 2–6 years and those aged ≥12 years (17.5%, *n* = 118; 14.9%, *n* = 81; and 12.6%, *n* = 89, respectively; *p* = 0.032). SRBDs were equally suspected among females and males in 2–6-year-old children (*p* = 0.393) as well as 7–11-year-old (*p* = 0.503) and ≥12-year-old children (*p* = 0.818).

According to the survey, 4.9% (*n* = 94) of parents reported that their children snored for more than half the night, 13.1% (*n* = 252) observed loud breathing, 5% (*n* = 97) noted repeated stops of breathing during sleep, and 22.2% (*n* = 426) reported EDS in their children. Nocturnal enuresis was observed in 4.1% (*n* = 62) of the children older than 5 years. Morning headaches were observed in 39.1% (*n* = 754) of the children. All these complaints were more commonly observed in children within the suspected SRBD group (Table 1). Snoring and repeated breathing stops during sleep had the highest odds ratios for suspected SRBDs (OR 10.979, 95% CI 7.088–17.006, *p* < 0.001 and OR 12.297, 95% CI 7.948–19.027, *p* < 0.001, respectively). It was noted that 48.0% (*n* = 926) of the children slept alone in their room at night. The prevalence of sleeping alone increased with age: 24.1% (*n* = 131) of children aged 2–6 years, 46.0% (*n* = 311) of children aged 7–11 years, and 68.3% (*n* = 484) of children aged ≥12 years were sleeping alone. SRBDs were more commonly suspected in co-sleeping children aged 7–11 years than in children of the same age who slept alone (20.6%, *n* = 75 and 13.8%, *n* = 43, respectively, *p* = 0.021). A similar pattern was observed in co-sleeping children aged 12 years or older (16.4%, *n* = 37 and 10.7%, *n* = 52, respectively; *p* = 0.033).

Overweight was observed in 18.5% (*n* = 357) of Lithuanian children, whereas obesity was found in 6.6% (*n* = 128). Overall, 20.4% of overweight children and 23.4% of obese children were suspected of having SRBDs in the study. Overweight was a significant risk factor for suspected SRBDs in the study population (*p* = 0.002), especially in children aged 7–11 years (*p* = 0.004) and ≥12 years (*p* = 0.015). Obesity was also a significant risk factor (*p* = 0.006). In age group analysis, obesity was associated with an increased risk of suspected SRBDs in children aged 7–11 years (*p* = 0.040), although no significant difference was found in those aged ≥12 years. Among children with SRBDs, obesity was not associated with EDS (*p* = 0.671).

Allergic rhinitis (AR) was a risk factor for suspected SRBDs in preschoolers (*p* = 0.029). Enlarged tonsils and adenoids (*p* < 0.001), attention-deficit/hyperactivity disorder (ADHD; *p* < 0.001), and endocrine diseases (*p* < 0.001) were significant risk factors for suspected SRBDs and persisted across all age groups. Tonsillectomy or adenoidectomy was performed more commonly in children with suspected SRBDs (*p* = 0.011), especially in those aged 7–11 years (*p* = 0.002). We did not find significant differences in the incidence of asthma (*p* = 0.102), cardiovascular diseases (*p* = 0.107), or neuromuscular diseases (*p* = 0.164) within our study population. The risk factor analysis of different age groups is presented in Table 2.

**Figure 1:**
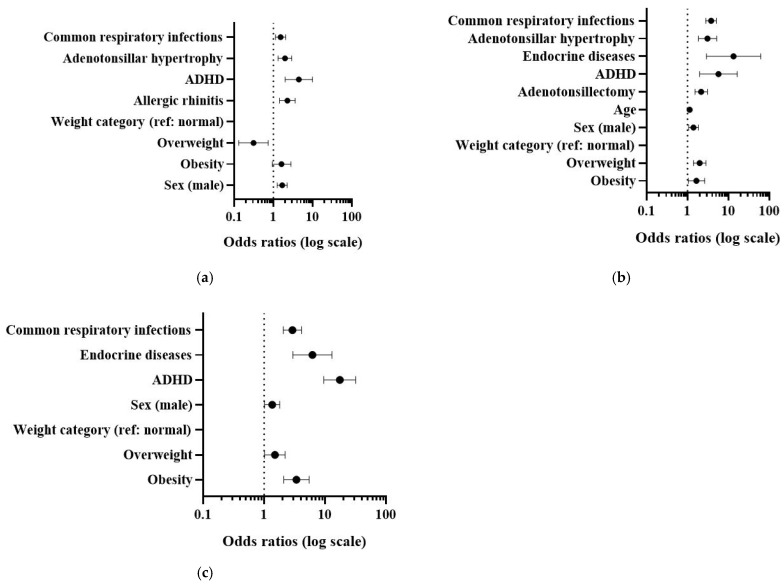
Multivariate logistic regression analysis showing adjusted odds ratios (95% confidence intervals) for risk factors of suspected SRBDs in children, stratified by age group: (**a**) Children aged 2–6 years, (**b**) children aged 7–11 years, and (**c**) children aged ≥12 years. The vertical dashed line indicates odds ratio of 1.0 (no association).

Multivariable binary logistic regression analysis was performed separately for each age group to identify factors independently associated with suspected SRBDs. Multicollinearity was assessed across all regression models. No evidence of problematic multicollinearity was observed overall. However, a higher condition index was identified in the 7–11-year age group model, although no substantial collinearity between independent variables was detected. This finding is likely attributable to the low prevalence of endocrine diseases in this subgroup. The detailed results of all three models are presented in Figure 1 and Supplementary Material S2.

In children aged 2–6 years, mental disorders showed the strongest association with suspected SRBDs (OR 4.46, 95% CI 1.99–9.97), followed by allergic rhinitis (OR 2.27, 95% CI 1.43–3.59) and adenotonsillar hypertrophy (OR 1.97, 95% CI 1.32–2.95) (Figure 1a). The Hosmer–Lemeshow goodness-of-fit test indicated acceptable model fit (*p* = 0.228). The model correctly classified 64.6% of cases (sensitivity 63.2%, specificity 66.1%) using a cut-off value of 0.50. ROC analysis demonstrated moderate model discrimination (AUC 0.681, 95% CI 0.645–0.718, *p* < 0.001).

In children aged 7–11 years, endocrine diseases demonstrated the strongest association with suspected SRBDs (OR 13.37, 95% CI 2.91–61.31), followed by ADHD (OR 5.69, 95% CI 1.98–16.36). Frequent respiratory infections (OR 3.77, 95% CI 2.80–5.08) and adenotonsillar hypertrophy (OR 3.08, 95% CI 1.84–5.16) were also independently associated with increased odds of suspected SRBDs (Figure 1b). The Hosmer–Lemeshow goodness-of-fit test indicated acceptable model fit (*p* = 0.054). The multivariable model showed acceptable discriminatory ability (AUC 0.745, 95% CI 0.716–0.775, *p* < 0.001) and correctly classified 67.8% of cases, with a sensitivity of 70.9% and specificity of 63.8%.

Among adolescents, ADHD demonstrated the strongest association with suspected SRBDs (OR 17.51, 95% CI 9.60–31.96), followed by endocrine diseases (OR 6.21, 95% CI 2.97–13.02) and obesity (OR 3.40, 95% CI 2.11–5.49) (Figure 1c). The Hosmer–Lemeshow goodness-of-fit test indicated acceptable model fit (*p* = 0.095). The multivariable model showed good discriminatory ability, with an AUC of 0.760 (95% CI 0.730–0.790, *p* < 0.001). Using a cut-off value of 0.50, the model correctly classified 71.1% of cases, with a sensitivity of 64.7% and specificity of 77.0%.

## 4. Discussion

This is the first study conducted in a Baltic state to assess the prevalence of children at risk for SRBDs and potential risk factors of SRBDs among 2–18-year-old children. The wide range of reported SRBD prevalence across various countries, along with the global health disparities, highlights the importance of this study’s findings. This study included children from various regions in Lithuania.

Approximately 15% of the children in our study were suspected of having SRBDs, and 21.9% of them reported snoring more than half the night. Various studies have reported the prevalence of SRBDs to be in the range of 0.7–13% in the general population of children [1,7,9,14]. A higher prevalence of OSA (up to 20%) has been reported in habitually snoring children [14]. It is important to note that SRBD testing declined significantly during the COVID-19 pandemic, and this study was conducted in the post-pandemic period. An analysis of children’s health in Lithuania during the COVID-19 pandemic revealed decreased physical activity, impaired sleep quality, and increased prevalence of overweight and obesity [15], which may have contributed to the slightly higher prevalence of children at risk for SRBDs in the country. Additionally, the prevalence of SRBDs was estimated using a translated and validated PSQ, which has a reported sensitivity of 72.7% and specificity of 64.6% for diagnosing moderate-to-severe sleep apnea [13]. Therefore, the observed prevalence reflects children at risk for SRBDs rather than clinically confirmed cases. These factors may explain why the prevalence observed in our study is at the higher end of the range reported in the literature.

In epidemiological studies focusing on adults, SRBDs have been found to have a higher prevalence in men than in women [16]. Although some pediatric studies have reported a male predominance regarding SRBDs, the overall evidence remains insufficient to confirm significant sex-related differences in children [2,16,17]. In our study, male sex was not identified as a significant risk factor for suspected SRBDs in the overall analysis of Lithuanian children. However, the logistic regression models revealed a positive association between male sex and an increased SRBD risk across all age groups.

In our study, SRBDs were more commonly suspected in school-aged children than in preschool-aged children or adolescents. One of the most common causes of OSA is an increased amount of lymphatic tissue in the upper airways, predominantly observed in children aged 2–8 years [3,5,18]. Meanwhile, obesity-related SRBDs are mostly observed in adolescents and are the second most common cause of SRBDs in children [1,3,4,18]. In our study population, overweight and obesity were most commonly identified in children aged 7–11 years old. Additionally, 12.8% of children with suspected SRBDs in this age group presented with enlarged tonsils and adenoids, which may contribute to upper airway obstruction. Consequently, a higher prevalence of suspected SRBDs was observed among children aged 7–11 years.

Snoring is the most commonly reported symptom of OSA, occurring in 8–27% of children [5,14,19]. Aubertin et al. (French consensus for management of pediatric OSA, 2023) reported that snoring and observed apneas during the night are the major criteria for suspecting OSA in children [20]. In our study, both symptoms were strongly associated with SRBDs in children. In some cases, habitual snoring is not accompanied by respiratory events, sleep arousals, or gas exchange abnormalities; in such instances, it is classified as primary snoring [2]. However, distinguishing between primary snoring and more serious conditions is clinically challenging [2,19]. Importantly, the PSQ is a screening tool that captures a spectrum of SRBD symptoms rather than providing a definitive diagnosis. Although snoring-related items are included in the PSQ, suspected SRBDs are determined based on the cumulative score, requiring multiple positive responses rather than a single symptom. Notably, compared with data in the literature, snoring was reported less frequently in our study population. We hypothesized that this may be due to symptoms being overlooked in children who sleep alone. The significantly higher prevalence of suspected SRBDs observed in co-sleeping children in our study suggests that SRBD symptoms are more likely to be noticed when family members sleep nearby.

In our study, EDS was a significant symptom of suspected SRBDs across all age groups. Moreira et al. reported a prevalence of EDS ranging from 4.0% to 6.6% in school-aged children [21]. Merdad et al. discussed various causes of EDS in adolescents, including OSA [22]. Although EDS in children with OSA is generally less pronounced than in adults, it remains one of the most significant daytime symptoms of SRBDs and is frequently included in various questionnaires designed to identify SRBDs [1,5,21]. Moreover, it is well known that children with OSA and obesity develop EDS more often than children without obesity [23]. This association is attributed to the increased release of sleepiness-promoting compounds, driven by systemic inflammation resulting from both obesity and OSA [23]. However, in our study, EDS was not associated with being overweight or obese.

In the literature, adenotonsillar hypertrophy is identified as the most common risk factor for OSA among children, particularly in preschool-aged children [1,3,5,18]. Although an increase in lymphatic tissue in the upper airways can cause a reduction of airflow, no significant association has been found between tonsillar size and OSA severity [2,5]. However, the assessment of adenotonsillar hypertrophy size is inherently subjective and may not always be the sole determining factor for OSA development. Craniofacial anomalies are also considered to play a crucial role in the etiology of OSA [1,2,3]. In their systematic review and meta-analysis, Finke et al. identified several cephalometric indicators relevant to OSA, including a thicker and longer soft palate, an increased distance from the hyoid bone to the mandible, a posterior mandibular rotation, and an enlarged upper anterior facial height [24]. However, the etiology of childhood OSA is multifactorial rather than caused by a single factor [3,5,25]. In our study, enlarged tonsils and adenoids were significant risk factors for suspected SRBD in preschool- and school-aged children. Additionally, we observed that children aged 7–11 years who had undergone tonsillectomy or adenoidectomy were more likely to be suspected of having SRBDs, suggesting that a substantial number of children may experience residual OSA. The American Academy of Sleep Medicine recommends performing PSG after adenotonsillectomy to assess residual OSA in children with preoperative evidence of moderate-to-severe OSA [8]. However, it is important to note that children in Lithuania remain inadequately screened for SRBDs, particularly before surgery.

Common respiratory tract infections were associated with suspected SRBDs across all age groups, with the strongest association observed in children aged 7 years and older. Persistent infections of the upper airway lymphoid tissue can lead to significant hypertrophy, which contributes to obstructive SRBDs [26]. Besedovsky et al. highlighted the bidirectional relationship between sleep and immunity, noting that adequate sleep duration not only improves infection outcomes but is also associated with a reduced risk of infectious diseases [27]. Taken together, these findings suggest a complex interplay between recurrent infections, immune function, and SRBDs.

In our study, AR was identified as a risk factor for suspected SRBDs in children aged 2–6 years. AR is commonly associated with snoring and disturbed sleep [3]. Although AR can sustain nasal mucosal inflammation, increase airway resistance, and promote oral breathing, there is still insufficient evidence to confirm that it directly causes OSA in children [2,3,25]. However, considering that preschool-aged children naturally have narrower upper airways, AR may be a contributing factor exacerbating SRBD symptoms [28].

Obesity is one of the most critical risk factors for OSA in both children and adults [25]. It is also associated with a high risk of residual OSA following adenotonsillectomy [2]. According to the literature, up to 60% of children with obesity experience OSA [4,29]. In our study, the prevalence of SRBDs among children with overweight and obesity was 20.4% and 23.4%, respectively. It has been reported that for each increase of 1 kg/m^2^ in BMI above the average, the risk of OSA increases by 12% [3]. In our study, obesity was identified as a significant risk factor for suspected SRBDs in children aged 6–11 years, whereas no such association was observed in preschool-aged children or adolescents. Notably, in the 2–6-year age group, overweight was associated with lower odds of SRBDs in the regression model, which is an unexpected finding and inconsistent with the existing literature. This result may reflect residual confounding or age-specific anatomical and physiological differences and should therefore be interpreted with caution. However, logistic regression models demonstrated that being overweight was a significant risk factor for suspected SRBDs in school-aged children and adolescents, whereas obesity was consistently associated with an increased risk across all age groups. Additionally, we found a significant association between suspected SRBDs and endocrine disorders. Thomas et al. discussed the bidirectional relationship between obesity and OSA [30]. In other cases, endocrine disorders such as increased insulin resistance, growth failure, and hypothyroidism have been reported as sequelae of untreated SRBDs [30]. Our study findings are consistent with the previous literature, although their interpretation is limited by the low number of cases and potential confounding factors.

The overlap between ADHD symptoms and OSA has been widely discussed in the literature, with attention deficits reported in up to 95% of pediatric patients [31]. Notably, one of the three scales used in the PSQ is the behavioral scale, which includes six questions specifically related to ADHD [11]. Therefore, the observed association between ADHD and suspected SRBDs in this study may be partially influenced by overlapping constructs within the PSQ, and should be interpreted with caution. At the same time, it is well established that sleep disruptions caused by intermittent hypoxia and hypercapnia during SRBDs significantly affect children’s cognitive function, often resulting in inattention [32]. Additionally, the elevated levels of inflammatory cytokines associated with SRBDs may contribute to poor cognitive function and attention deficits [32]. In our study, ADHD was reported by a few children and was significantly associated with suspected SRBDs across all age groups. In logistic regression models, ADHD consistently demonstrated a high OR across all age groups, with the strongest association observed in adolescents, suggesting its significant role as a risk factor for SRBDs. These findings suggest a notable connection between suspected SRBDs and cognitive or behavioral difficulties, highlighting the importance of recognizing and addressing SRBDs in children to support their cognitive and emotional well-being.

Overall, our findings demonstrate that the pattern of SRBD-associated factors shifts with age, with inflammatory upper airway conditions predominating in younger children and metabolic or neurobehavioral factors becoming more prominent in older age groups.

This study has several important strengths. First, it is based on a large sample size, which increases the statistical power of the analyses and enhances the reliability of the findings. Second, to our knowledge, this is the first large-scale study investigating children at risk for SRBDs in the Baltic region, providing novel epidemiological data in an area where such information has been limited. Additionally, the study includes a detailed analysis of SRBD risk factors, allowing for the identification of the proportion of children at risk and the characterization of the most prevalent risk factors in the region. These findings offer valuable insights for regional healthcare planning and may contribute to the development of targeted screening and triage strategies, with particular attention to obese adolescents and younger children with adenotonsillar hypertrophy, who may represent clinically relevant higher-risk groups.

This study also has several limitations. First, it was based on voluntary parental participation, which may have influenced the representativeness of the sample. Families with higher awareness of sleep disorders or health-related concerns may have been more likely to participate, introducing a potential selection bias. Furthermore, although educational institutions from all Lithuanian regions were included, certain demographic groups, such as children from rural areas or those whose families lacked digital literacy or access to online surveys, may have been underrepresented. These factors could have contributed to sampling bias and limited the generalizability of the findings. Second, because health condition data were based solely on parental reports, the study may be affected by classification bias. Additionally, BMI was calculated using parent-reported height and weight, which may be subject to reporting inaccuracies. This limitation may have led to misclassification, potentially influencing the observed associations with SRBDs. Furthermore, we used the PSQ to identify suspected SRBDs instead of PSG, which is considered the gold standard for diagnosing SRBDs. Although the PSQ is a validated screening tool, it does not offer the same level of diagnostic accuracy as PSG, particularly in detecting mild sleep apnea. This may have resulted in misclassification and limited the precision of the findings. Future prospective studies that include PSG are necessary to provide more accurate results on the prevalence of SRBDs and their risk factors, which are crucial for developing an effective triage system. This is particularly important in regions where access to PSG is limited, ensuring that resources are prioritized for children at the highest risk.

## 5. Conclusions

The prevalence of suspected SRBDs in the general pediatric population of Lithuania is approximately 15%. School-aged children were identified as the group at the highest risk. In preschool-aged children, AR and adenotonsillar hypertrophy were the predominant associated factors. Among school-aged children, endocrine diseases showed the strongest association, followed by adenotonsillar hypertrophy, while overweight and obesity were also significant. In adolescents, endocrine diseases and obesity were most strongly associated with suspected SRBDs. Across all age groups, ADHD and common respiratory tract infections remained consistently associated with suspected SRBDs. These findings underscore the age-dependent pattern of SRBD-associated factors and support the need for age-specific risk assessment and early identification strategies in pediatric populations.

## Figures and Tables

**Table 1:** Clinical and Demographic Characteristics of Patients.

	Suspected SRBD Group, % or Median, (*n* or 95% CI)	Control Group, % or Median, (*n* or 95% CI)	OR (95% CI)	*p*-Value
**Median age**	9 (8.82–9.77)	10 (9.50–9.93)	-	0.142
**Female**	47.2 (136)	49.5 (810)	-	0.485
**Median BMI**	17.12 (18.18–18.56)	17.09 (17.60–17.99)	-	0.072
**Sleeps not alone in the room**	57.1 (164)	51.0 (837)	-	0.056
**Infectious respiratory diseases per year**	3 (3.36–4.07)	2 (2.85–3.09)	-	**<0.001**
2–6 y/o	5 (4.46–6.17)	4 (4.27–4.80)	-	0.189
7–11 y/o	3 (2.92–3.83)	2 (2.41–2.74)	-	**<0.001**
≥12 y/o	2 (2.77–3.27)	2 (2.04–2.36)	-	**0.011**
**Snoring**	21.9 (56)	2.5 (38)	10.98 (7.09–17.01)	**<0.001**
2–6 y/o	26.6 (20)	3.3 (15)	9.95 (4.83–20.52)	**<0.001**
7–11 y/o	12.6 (25)	1.7 (9)	17.83 (8.04–39.57)	**<0.001**
≥12 y/o	15.3 (11)	2.5 (14)	6.92 (3.01–15.91)	<0.001 *
**Loudly breathing**	45.8 (124)	8.3 (128)	9.35 (6.93–12.61)	**<0.001**
2–6 y/o	49.4 (39)	10.6 (48)	8.21 (4.82–13.98)	**<0.001**
7–11 y/o	46.0 (52)	7.1 (38)	11.15 (6.79–18.30)	**<0.001**
≥12 y/o	41.8 (33)	7.5 (42)	8.83 (5.11–15.26)	**<0.001**
**Noticed stops of breathing**	23.8 (60)	2.5 (37)	12.30 (7.95–19.03)	**<0.001**
2–6 y/o	25.3 (19)	3.2 (14)	10.25 (4.87–21.58)	<0.001 *
7–11 y/o	26.4 (28)	3.3 (17)	10.45 (5.37–19.99)	**<0.001**
≥12 y/o	18.3 (13)	1.1 (6)	20.10 (7.36–54.88)	<0.001 *
**Excessive daytime sleepiness**	55.6 (155)	17.1 (271)	6.04 (4.61–7.92)	**<0.001**
2–6 y/o	35.9 (28)	7.0 (32)	7.40 (4.12–13.30)	**<0.001**
7–11 y/o	52.6 (60)	9.8 (53)	10.23 (6.43–16.28)	**<0.001**
≥12 y/o	77.0 (67)	31.8 (186)	7.19 (4.24–12.20)	**<0.001**
**Nocturnal enuresis**	19.8 (56)	8.1 (132)	2.79 (1.98–3.92)	**<0.001**
2–6 y/o	46.8 (37)	22.8 (103)	2.98 (1.82–4.88)	**<0.001**
7–11 y/o	14.5 (17)	4.0 (22)	4.10 (2.10–7.99)	**<0.001**
≥12 y/o	2.3 (2)	1.1 (7)	-	0.306
**Morning headache**	73.6 (209)	33.6 (545)	5.52 (4.17–7.32)	**<0.001**
2–6 y/o	64.6 (51)	36.7 (168)	3.14 (1.91–5.18)	**<0.001**
7–11 y/o	71.8 (84)	32.8 (181)	5.22 (3.36–8.10)	**<0.001**
≥12 y/o	84.1 (74)	31.9 (196)	11.27 (6.21–20.46)	**<0.001**

CI—confidence interval; OR—odds ratio; SRBD—sleep-related breathing disorder. * Fisher’s exact test used.

**Table 2:** Risk factors analysis for suspected SRBDs in the study population.

	Suspected SRBD Group, % (*n*)	Control Group, % (*n*)	OR (95% CI)	*p*-Value
**Overweight** **(BMI ≥ 85th ‰)**	25.7 (73)	17.8 (284)	1.60 (1.19–2.15)	**0.002**
2–6 y/o	10.9 (8)	12.8 (58)	-	0.487
7–11 y/o	33.3 (39)	20.9 (113)	1.89 (1.22–2.93)	**0.004**
≥12 y/o	29.9 (26)	18.7 (113)	1.85 (1.12–3.06)	**0.015**
**Obesity (BMI ≥ 95th ‰)**	10.6 (30)	6.1 (98)	1.81 (1.18–2.78)	**0.006**
2–6 y/o	8.8 (7)	6.4 (29)	-	0.440
7–11 y/o	12.0 (14)	6.5 (35)	1.97 (1.02–3.78)	**0.040**
≥12 y/o	10.3 (9)	5.6 (34)	-	0.088
**Enlarged tonsils and adenoids**	12.4 (35)	6.2 (100)	2.13 (1.42–3.20)	**<0.001**
2–6 y/o	20.3 (16)	10.7 (49)	2.11 (1.13–3.94)	**0.017**
7–11 y/o	12.8 (15)	4.8 (26)	2.92 (1.50–5.72)	**0.001**
≥12 y/o	4.6 (4)	4.1 (25)	-	0.503 *
**Performed tonsillectomy or adenoidectomy**	19.5 (56)	13.8 (225)	1.42 (1.09–1.85)	**0.011**
2–6 y/o	9.9 (8)	5.9 (27)	-	0.176
7–11 y/o	23.7 (28)	12.8 (71)	1.86 (1.26–2.74)	**0.002**
≥12 y/o	22.7 (20)	20.5 (127)	-	0.633
**Common infectious respiratory diseases**	44.1 (93)	25.7 (361)	2.28 (1.69–3.07)	**<0.001**
2–6 y/o	57.9 (33)	46.7 (191)	-	0.113
7–11 y/o	47.7 (41)	19.3 (92)	3.81 (2.36–6.16)	**<0.001**
≥12 y/o	27.9 (19)	15.0 (78)	2.19 (1.23–3.92)	**0.007**
**Allergic rhinitis**	14.1 (40)	10.2 (164)	-	0.050
2–6 y/o	15.2 (12)	7.7 (35)	2.15 (1.07–4.26)	**0.029**
7–11 y/o	9.4 (11)	8.3 (45)	-	0.695
≥12 y/o	19.5 (17)	13.8 (84)	-	0.156
**Asthma**	5.7 (16)	3.6 (58)	-	0.102
2–6 y/o	6.3 (5)	2.9 (13)	-	0.111 *
7–11 y/o	5.1 (6)	3.9 (21)	-	0.341 *
≥12 y/o	5.7 (5)	3.9 (24)	-	0.292 *
**ADHD**	10.6 (30)	1.7 (28)	6.68 (3.93–11.38)	**<0.001**
2–6 y/o	6.3 (5)	1.8 (8)	3.78 (1.21–11.88)	0.003 *
7–11 y/o	6.0 (7)	0.7 (4)	8.58 (2.47–29.79)	<0.001 *
≥12 y/o	20.7 (18)	2.6 (16)	9.65 (4.71–19.79)	<0.001 *
**Endocrine diseases**	3.9 (11)	0.7 (12)	5.38 (2.35–12.31)	<0.001 *
**Cardiovascular diseases**	3.2 (9)	1.7 (28)	-	0.107
**Neuromuscular diseases**	0.7 (2)	0.2 (3)	-	0.164 *

ADHD—attention-deficit/hyperactivity disorder; BMI—body mass index; CI—confidence interval; OR—odds ratio; SRBD—sleep-related breathing disorder. * Fisher’s exact test used.

## Data Availability

The data that support the findings of this study are available from the corresponding author upon reasonable request.

## Supplementary Materials

The following supporting information can be downloaded at https://www.mdpi.com/article/10.3390/medicina62040707/s1: Supplementary Material S1: The detailed survey questionnaire used in this study. Supplementary Material S2: Results of the binary regression analysis identifying risk factors for suspected SRBDs in children of different age groups.

## Abbreviations

The following abbreviations are used in this manuscript:

ADHD	Attention-deficit/hyperactivity disorder
AR	Allergic rhinitis
BMI	Body mass index
CI	Confidence intervals
EDS	Excessive daytime sleepiness
OR	Odds ratio
OSA	Obstructive sleep apnea
PSG	Polysomnography
PSQ	Pediatric Sleep Questionnaire
SRBD	Sleep-related breathing disorder
