# Supplementary material for: Age-Dependent Risk Factors in Pediatric Sleep-Disordered Breathing: A Large-Scale Cross-Sectional Study
Source: Medicina (Kaunas). 2026 Apr 7;62(4):707. doi: 10.3390/medicina62040707 (PMC13117163; doi:10.3390/medicina62040707)
Supplement: Supplementary file 1 [file medicina-62-00707-s001.zip › Supplementary Material S2.pdf]

Supplementary Material S2. Results of the binary regression analysis identifying risk factors for suspected SRBD in children of different age groups.

	B	p-value	OR	95% CI
		**2–6 years old children**		
**Common respiratory infections**	0.42	0.005	1.52	1.13–2.06
**Adenotonsillar hypertrophy**	0.68	<0.001	1.97	1.32–2.95
**ADHD**	1.49	<0.001	4.46	1.99–9.97
**Allergic rhinitis**	0.82	<0.001	2.27	1.43–3.59
**Weight category**		0.005		
**Overweight**	-1.17	0.008	0.31	0.13–0.74
**Obesity**	0.48	0.090	1.61	0.93–2.79
**Sex (male)**	0.52	<0.001	1.68	1.25–2.25
**Constant**	-0.80	<0.001		
		**7–11 years-old children**		
**Common respiratory infections**	1.33	<0.001	3.77	2.80–5.08
**Adenotonsillar hypertrophy**	1.13	<0.001	3.08	1.84–5.16
**Endocrine diseases**	2.59	<0.001	13.37	2.91–61.31
**ADHD**	1.74	0.001	5.69	1.98–16.36
**Adenotonsillectomy**	0.77	<0.001	2.16	1.52–3.07
**Age**	0.12	0.012	1.13	1.03–1.24
**Sex (male)**	0.34	0.016	1.40	1.07–1.84
**Weight category**		<0.001		
**Overweight**	0.68	<0.001	1.98	1.40–2.81
**Obesity**	0.50	0.034	1.65	1.040–2.62
**Constant**	-2.00	<0.001		
		**⩾12 years-old children**		
**Common respiratory infections**	1.07	<0.001	2.93	2.07–4.13
**Endocrine diseases**	1.83	<0.001	6.21	2.97–13.02
**ADHD**	2.86	<0.001	17.51	9.60–31.96
**Sex, male**	0.31	0.036	1.36	1.02–1.80
**Weight category**		<0.001		
**Overweight**	0.41	0.038	1.51	1.02–2.22
**Obesity**	1.22	<0.001	3.40	2.11–5.49
**Constant**	-1.06	<0.001		
