# Supplementary material for: Age-Dependent Risk Factors in Pediatric Sleep-Disordered Breathing: A Large-Scale Cross-Sectional Study
Source: Medicina (Kaunas). 2026 Apr 7;62(4):707. doi: 10.3390/medicina62040707 (PMC13117163; doi:10.3390/medicina62040707)
Supplement: Supplementary file 1 [file medicina-62-00707-s001.zip › Survey_Supplementary material S1.pdf]

LITHUANIAN UNIVERSITY OF HEALTH SCIENCES

Dear Parents or Guardians,

We represent the Department of Pediatrics at the Lithuanian University of Health Sciences. We are conducting a national study titled *"The Prevalence of Symptoms Indicative of Sleep-Related Breathing Disorders Among Children in Lithuania."* As part of this research, we are inviting parents or legal guardians of children aged 2 to 17 to participate in a survey about their child's health and sleep.

The aim of the study is to assess the prevalence of sleep-related breathing disorders using the Pediatric Sleep Questionnaire and to analyze its association with potential risk factors in the general pediatric population of Lithuania. The questionnaire is anonymous. Based on the responses provided, it will not be possible to identify individual participants. All data collected during the study will remain confidential and will be used solely for scientific purposes. Please mark or write the answers that best reflect your opinion.

□ I confirm that I agree to participate in this study. I understand that my participation is voluntary and that all data collected will remain anonymous. I give my consent for the provided responses to be used for scientific research purposes.
□ I do not agree to participate in this study.

I part

1. **Please enter your child's age**:_____________
2. **Child's gender (please select):**
   - ○ Male
   - ○ Female
   - ○ Other.
3. **Please enter:**
4. **Your child's height (cm):** ____________
5. **Your child's weight (kg):** ____________
6. **Do you sleep in the same bed with your child?**
   - ○ Yes
   - ○ No
7. **How many people sleep in the same room as your child?**
   - ○ The child sleeps alone
   - ○ One
   - ○ Two
   - ○ More than two
8. **How many times has your child had a cold or upper respiratory infection in the past year? (please specify)** ___________________

II part. Please select the most appropriate answer about your child.

1. Please indicate if your child has any of the following conditions:

- □ Frequent colds
- □ Enlarged tonsils and adenoids
- □ Allergic rhinitis
- □ Asthma
- □ Cardiovascular diseases (congenital heart defects, arterial hypertension, arrhythmias, other conditions)
- □ Endocrine disorders (diabetes, thyroid disorders, other conditions)
- □ Mental health disorders (attention-deficit/hyperactivity disorder)
- □ Neuromuscular disorders (spinal muscular atrophy, Duchenne syndrome, etc.)
- □ I prefer not to provide information about my child's health
- □ My child is healthy.

2. Has your child previously undergone a tonsillectomy or adenoidectomy?

- o Yes
- o No

3. How many hours per day does your child sleep?

- o Less than 6 hours
- o 6–8 hours
- o 9–12 hours
- o More than 12 hours

4. What time does your child usually go to bed on weekdays?

- o 8:00–9:00 PM
- o 9:00–10:00 PM
- o 10:00–11:00 PM
- o 11:00 PM–12:00 AM
- o After midnight

5. What time does your child usually go to bed on weekends?

- o 8:00–9:00 PM
- o 9:00–10:00 PM
- o 10:00–11:00 PM
- o 11:00 PM–12:00 AM
- o After midnight

6. Before bedtime, your child usually:

- □ Uses a phone
- □ Watches TV
- □ Plays computer/video games
- □ Engages in physical activity
- □ Eats or snacks
- □ Spends time calmly
- □ Other

III part: Pediatric Sleep Questionnaire. Please select the most appropriate answer about your child.

While sleeping does your child:

Q1 Snore more than half the time:
- Yes
- No
- I don’t know

Q2 Always snore:
- Yes
- No
- I don’t know

Q3 Snore loudly:
- Yes
- No
- I don’t know

Q4 Have “heavy” or loud breathing:
- Yes
- No
- I don’t know

Q5 Have trouble breathing, or struggle to breath:
- Yes
- No
- I don’t know

Q6 Have you ever seen your child stop breathing during the night:
- Yes
- No
- I don’t know

Does your child:

Q7 Tend to breathe through the mouth during the day:
- Yes
- No
- I don’t know

Q8 Have a dry mouth on waking up in the morning:
- Yes
- No
- I don’t know

Q9 Occasionally wet the bed:
- Yes
- No
- I don’t know

Q10 Wake up feeling unrefreshed in the morning:

- Yes
- No
- I don’t know

Q11 Have a problem with sleepiness during the day:

- Yes
- No
- I don’t know

Q12 Has a teacher commented that your child appears sleepy during the day:

- Yes
- No
- I don’t know

Q13 It is hard to wake your child up in the morning:

- Yes
- No
- I don’t know

Q14 Does your child wake up with headaches in the morning:

- Yes
- No
- I don’t know

Q15 Did your child stop growing at a normal rate at any time since birth:

- Yes
- No
- I don’t know

Q16 Is your child overweight:

- Yes
- No
- I don’t know

Your child often…

Q17 Does not seem to listen when spoken to directly

- Yes
- No
- I don’t know

Q18 Has difficulty organizing tasks and activities

- Yes
- No
- I don’t know

Q19 Is easily distracted by extraneous stimuli

- Yes
- No
- I don’t know

Q20 Fidgets with hands or feet or squirms in seat

- Yes
- No
- I don’t know

Q21 Is “on the go” or often acts as if “driven by a motor”

- Yes
- No
- I don’t know

Q22 Interrupts or intrudes on others

- Yes
- No
- I don’t know

Thank you for your responses and for participating in the study!
